# The NLRP3 inflammasome is involved in resident intruder paradigm-induced aggressive behaviors in mice

**DOI:** 10.3389/fphar.2023.974905

**Published:** 2023-01-25

**Authors:** Qingying Yu, Molin Liu, Weibo Dai, Yu Xiong, Xiangyu Mu, Mengyao Xia, Yanling Li, Shan Ma, Yongtao Su, Jibiao Wu, Chuanguo Liu, Yicheng Xie, Tingting Zhao, Aimei Lu, Ning Weng, Feng Zheng, Peng Sun

**Affiliations:** ^1^ School of Pharmacy, Shandong University of Traditional Chinese Medicine, Ji’nan, China; ^2^ Department of Pharmacy, Zhongshan Hospital of Traditional Chinese Medicine, Zhong Shan, China; ^3^ Department of Neurosurgery, The Second Affiliated Hospital of Fujian Medical University, Quanzhou, China; ^4^ College of Traditional Chinese Medicine, Shandong University of Traditional Chinese Medicine, Ji’nan, China; ^5^ School of Rehabilitation Medicine, Shandong University of Traditional Chinese Medicine, Ji’nan, China; ^6^ Innovation Research Institute of Chinese Medicine, Shandong University of Traditional Chinese Medicine, Ji’nan, China; ^7^ Experimental center, Shandong University of Traditional Chinese Medicine, Ji’nan, China; ^8^ The Children’s Hospital, Zhejiang University School of Medicine, National Clinical Research Center for Child Health, Hangzhou, China; ^9^ College of Foreign Languages, Shandong University of Traditional Chinese Medicine, Ji’nan, China; ^10^ Shandong Public Health Clinical Center, Ji’nan, China; ^11^ Department of Traditional Chinese Medicine, Shandong Mental Health Center, Shandong University, Ji’nan, China

**Keywords:** NLRP3 inflammasome, aggressive behavior, resident-intruder paradigm, neuroplasticity, neural para-inflammation

## Abstract

**Background:** Aggressive behaviors are one of the most important negative behaviors that seriously endangers human health. Also, the central para-inflammation of microglia triggered by stress can affect neurological function, plasticity, and behavior. NLRP3 integrates stress-related signals and is a key driver of this neural para-inflammation. However, it is unclear whether the NLRP3 inflammasome is implicated in the development of aggressive behaviors.

**Methods:** First, aggressive behavior model mice were established using the resident intruder paradigm. Then, aggressive behaviors were determined with open-field tests (OFT), elevated plus-maze (EPM), and aggressive behavior tests (AT). Moreover, the expression of P2X7R and NLRP3 inflammasome complexes were assessed by immunofluorescence and Western blot. The levels of NLRP3 and inflammatory cytokines were evaluated using enzyme-linked immunosorbent assay (ELISA) kits. Finally, nerve plasticity damage was observed by immunofluorescence, transmission electron microscope, and BrdU staining.

**Results:** Overall, the resident intruder paradigm induced aggressive behaviors, activated the hippocampal P2X7R and NLRP3 inflammasome, and promoted the release of proinflammatory cytokines IL-1β in mice. Moreover, NLRP3 knockdown, administration of P2X7R antagonist (A804598), and IL-1β blocker (IL-1Ra) prevented NLRP3 inflammasome-driven inflammatory responses and ameliorated resident intruder paradigm-induced aggressive behaviors. Also, the resident intruder paradigm promoted the activation of mouse microglia, damaging synapses in the hippocampus, and suppressing hippocampal regeneration in mice. Besides, NLRP3 knockdown, administration of A804598, and IL-1Ra inhibited the activation of microglia, improved synaptic damage, and restored hippocampal regeneration.

**Conclusion:** The NLRP3 inflammasome-driven inflammatory response contributed to resident intruder paradigm-induced aggressive behavior, which might be related to neuroplasticity. Therefore, the NLRP3 inflammasome can be a potential target to treat aggressive behavior-related mental illnesses.

## Graphical Abstract

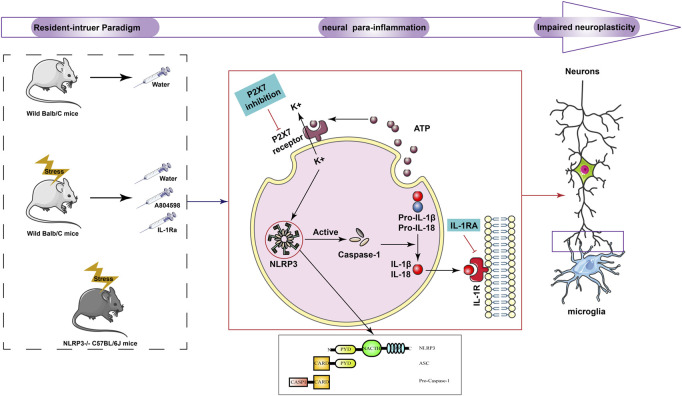

## 1 Introduction

Attacks are stress responses, manifested by collective external behavioral changes, emotional changes, and internal physiological changes in the body. When the body is not adapted to the stress response, abnormal behavioral responses can be manifested. On the other hand, attacks are essential to obtain and protect territory, food, partners, and future generations. However, repeated aggressive experiences can lead to compulsive pathological attacks. Some patients with mental disorders such as schizophrenia, antisocial personality disorder, and impulse control disorder can be harmful to society due to aggressive behaviors such as violent ones (Das et al., 2016). The Lancet’s article “No Health Without Mental Health” pointed out that “14% of the global disease burden comes from mental and nerve-related diseases, and mental abnormalities increase the risk of cardiovascular disease, diabetes, *tuberculosis*, and even infectious diseases and trauma” (Prince et al., 2007). For example, emotional stress can induce aggressive behaviors and aggravate arteriosclerosis, increasing the risk of coronary heart disease and arrhythmia (Peter et al., 2011). Escalation and abnormality of aggressive behaviors are common features of many mental illnesses and neurological diseases, but no effective treatment is currently available. Therefore, a deeper understanding of the pathogenic events and molecular changes that occur during aggressive behavior is required to allow the development of more effective treatments for these patients.

The causes of aggressive behavior are complex, including abnormalities in inflammatory markers, neurotransmitters, lipoproteins, and hormones (Sourav et al., 2016). In *postmortem* studies, Steiner et al. (2011); Liu et al. (2022) found a large number of activated microglia in the cingulate cortex and prefrontal cortex, and significantly elevated levels of inflammatory mediators in these regions in suicidal patients with depression, which suggests that there is an inflammatory state in the brain of patients with depression. Other studies have shown that long-term use of the neuroglia inhibitor minocycline decreases the number of polarized M1 microglia and increases the number of polarized M2 microglia in the CNS, which further led to a reduction in the expression of proinflammatory cytokine such as IL-1β, as well as an increase in the expression of anti-inflammatory cytokines such as IL-10. So, it may alleviating depressive-like behaviour in mice by suppressing CNS neuroinflammatory responses (Nnba et al., 2014). At the same time, psychosocial stress increases the body’s oxidative stress and inflammatory state (Patki et al., 2013). Also, intermittent explosive disorder patients have elevated plasma inflammatory markers, which can be accompanied by aggressive behaviors (CoccaroRoyce and Mary, 2014). Therefore, in recent years, many studies have evaluated the correlation between aggressive behaviors, immune cells, and inflammatory cytokines. In recent years, P2X7R antagonists in the treatment of mental diseases has also made progress. Antipsychotic drugs, such as chlorpromazine, haloperidol, can antagonize P2X7R mediated reactions, and reducing the activity of dopaminergic transmitter system by inhibiting P2X7R (He et al., 2017). Many studies also have demonstrated that the activation of NLRP3 inflammasome plays an important role in the development of depression (Alcocer-Gómez and Cordero, 2014; Kaufmann et al., 2017). Clinical studies found that NLRP3 gene expression and IL-1β in peripheral blood mononuclear immune cells of depression patients was increased (Alcocer-Gómez et al., 2014). Blocking NLRP3 reduced IL-1β level in hippocampus and alleviated depressive-like behavior (Alcocer-Gómez et al., 2016). Administration of P2X7 antagonists also blocked chronic stress-induced loss of pleasure and anxiety-like behavior (Masaaki et al., 2016). Although the etiological mechanism is not clear, these studies suggested that inflammatory biomarkers might be related to aggression.

Therefore, in the present study, we established an aggressive behavior mice model using the resident intruder paradigm and investigated the role of the NLRP3 inflammasome in aggressive behaviors. Our results showed that the resident intruder paradigm activated the NLRP3 inflammasome-driven inflammatory signaling pathway. Meanwhile, blockage of the NLRP3 inflammasome activation ameliorated resident intruder paradigm-induced aggressive behaviors.

## 2 Materials and methods

### 2.1 Chemicals

Chemicals are listed as follows: A804598 (HY-100483, MedChemExpress Co., Ltd. United States); IL-1Ra (HY-P7029A, MCE, United States); DMSO (D8370, Solarbio, China); Mouse NLRP3 ELISA kit (JYM0765Mo, Wuhan ColorfulGene biological technology Co., Ltd. China); Mouse IL-1β ELISA kit (JYM0531Mo, Wuhan ColorfulGene biological technology Co., Ltd. China); BCA Protein Assay Kit (PC0020, Solarbio, China); SDS-PAGE Gel Rapid Preparation Kit (G2037-50T, Servicebio, China); Recombinant Anti-beta Actin antibody (GB15003, Servicebio, China); NLRP3 Antibody (DF7438, Affinity Biologicals, Canada); Recombinant Anti-P2X7 antibody (ab259942, abcam, United Kingdom); HRP-conjugated goat anti-rabbit IgG (GB23204, Servicebio, China); Anti-Iba1 Rabbit pAb (GB11105, Servicebio, China); Cy3-labeled goat anti-rabbit IgG (GB21303, Servicebio, China); FITC-labeled goat anti-rabbit IgG (GB22303, Servicebio, China); Anti-BrdU Mouse mAb (GB12051, Servicebio, China); Anti-NeuN Rabbit pAb (GB11138, Servicebio, China); HE staining solution (G1005-100ML, Servicebio, China).

### 2.2 Animals and experimental design

Male C57BL/6 mice (5 weeks old, 13–17 g) and male BALB/C mice (7 weeks) were obtained from the Beijing Vital River Laboratory Animal Technology Co., Ltd., Male NLRP3^−/−^ mice (7 weeks old, 18–22 g) were obtained from THE JACKSON LABORATORY. Animals were kept under standard specific pathogen-free conditions at 22°C ± 2°C, 60% humidity and 12 h light/dark cycle. Food and water were available *ad libitum*. BALB/C and NLRP3^−/−^ mice were used as residents and C57BL/6J mice were used as invaders. Residents and invaders were fed separately with similar conditions (Haihong et al., 2010). The Ho HP (Melchior et al., 2004) research group used the Resident intruder paradigm to simulate an aggressive behavior rat model. The resident intruder paradigm-induced aggressive behavior mice model experiments were carried out in residential mice breeding environment at 14:30–17:30. Fifteen min before the start of the experiment, the invading mice were moved to the residential mice environment for adaptation. The resident mice and rearing cage were placed under the camera, food and water bottle were removed from the cage. Finally, the invading mice were placed in the cage for 10 min (Ho et al., 2001). After the shooting, the invading mice were taken out and put back into the original cage. Resident mice faced different invaders every day as designed by the Latin square. A residence invasion induction experiment was conducted on all residents for four consecutive days. After these behavioral experiments, the aggressive behavior score of the aggressive behavior test experiment was regarded as the main analysis index, then sorted in descending order. Mice with aggressive behavior scores ≥18 were included in the aggressive behavior model standard. These mice were randomly grouped according to aggressive behavior scores: behavior model mouse (Model), model + A804598 (A804598), and model + IL-1Ra (IL-1Ra), 10 mice per group. Mice with aggressive behavior scores close to zero were considered the blank control group (Control), and 10 NLRP3^−/−^ mice were used as the NLRP3^−/−^ control group (NLRP3^−/−^). The remaining mice were eliminated. After model mice screening and grouping, administration started and lasted for 7 days. A804598 (30 mg/kg) and IL-1Ra (3 mg/kg) were injected at 0.2 ml/10 g, and the other groups were injected with equal volume of saline. The administration was daily performed at 8:30. The experimental mice were subjected to behavioral testing 30 min after the last administration. The administration method was to gavage the drug to the second mouse in 15 min after the administration of the first mouse, and so on, which to ensure that each mouse was tested for behavior 30 min after the administration. Behavioral tests were conducted in the order of aggressive test, open field test and elevated plus maze test. All animal procedures were approved by the Committee for Experimental Animal Use and Care of Shandong University of Traditional Chinese Medicine.

### 2.3 Open-field test

The behavioral tests were performed at the end of A804598 or IL-1Ra treatments. The OFT has good reliability and validity (Chen et al., 2004), and using each behavior index score gradient change the animal model type can be identified (Katz et al., 1981). When the total distance of exercise significantly increases, a higher level of voluntary exercise behavior and a high level of exploratory activities can be determined. When the stay time in the central area significantly reduces, anxiety-like behavior can be determined (Keeney and Hogg, 1999). The open-field apparatus (50 × 50 × 40 cm) was divided into nine equal squares (Paulus et al., 1999). During the experiment, the surrounding environment was kept as quiet as possible. Before each trial, the field box was cleaned using 75% ethanol solution. All mice were left to adapt to the experimental environment for at least 1 hour. Then, they were placed in the middle of the field box. Mice activity was recorded for 6 min and analyzed by SuperMaze + high-throughput animal behavior experiment analysis software (Shanghai XinRuan Information Technology Co., Ltd., Shanghai, China). The data acquired included the percentage of residence time in the center, the percentage of travel distance in the center, the number of entries into the center, and the total distance traveled.

### 2.4 Elevated plus maze (EPM)

The EPM has advantages such as simplicity, speed, and sensitivity, being the most widely used classic anxiety behavior experiment in anti-anxiety research (Dalvi and Rodgers, 1999; Moraes et al., 2008). Since animals have the inquiring characteristics of the elevated and novel environment at the same time as they have the fear of hanging their arms high, they will face psychological contradictions and conflicts, which can be used to investigate their anxiety state. The ratio of the number of times the animal enters the open arm and the ratio of the stay time of the open arm are the classic indicators in this test (Barbalho et al., 2009). During the experiment, the surrounding environment was kept as quiet as possible. Before each trial, the maze was cleaned using 75% ethanol solution. All mice were allowed to acclimate to the experimental environment for at least 1 hour. Then, they were placed in the central platform facing one of the open arms. Mice were allowed to freely explore the maze for 5 min while being recorded for offline analyses. The entry into an arm was defined as when the animal moved all of its four paws into one arm. Results are expressed as the percentage of entries into the open arms (%) = (number of entries into the open arms/total number of entries into four arms) × 100%; and the percentage of time spent in the open arms (%) = (time spent in the open arms/total time spent in four arms) × 100%.

### 2.5 Aggressive behavior test

After the resident intruder paradigm induction experiment was over, the test mice started the aggressive behavior test at 14:30–17:30 on the next day. Invading mice were moved to the resident mice environment 15 min before the experiment begin. The resident mice and their rearing cage were placed under the camera, food and water bottle were removed from the cage. Finally, the invading mouse was placed in the cage for 10 min. After shooting, the invading mice were taken out and put back in the original cages. All the video materials were kept. After the aggressive behavior test, the aggressive behavior analysis tool was used to evaluate aggressive behaviors (Walburger et al., 2001).

### 2.6 Sample collection

#### 2.6.1 ELISA and WB sample collection methods

After behavioral experiment, four mice were anesthetized with isoflurane and blood samples were collected. After 30 min, the blood samples were centrifuged at 4°C, and the supernatant was stored in −80°C refrigerator. After taking blood, the mice were decapitated, and hippocampus were separated on ice and stored in −80°C refrigerator.

#### 2.6.2 HE staining, IHC and IF sample collection methods

Three mice were anesthetized with isoflurane and subjected to cardiac perfusion each group. After perfusion, the brains were decapitated and the brain samples were fixed in 4% paraformaldehyde in an EP tube and stored at room temperature.

#### 2.6.3 TEM sample collection methods

After the behavioral experiment, three mice were anesthetized with isoflurane for heart perfusion. Hippocampal was separated on ice and divided into 1 mm^3^ tissue blocks, which were placed in EP tubes with precooled glutaraldehyde and stored at 4°C.

### 2.7 Paraffin section making

1) The fresh tissue was fixed with 4% paraformaldehyde for more than 24 h. Put the trimmed tissue and the label in the dehydration box.
2) Dehydration and wax leaching: 75% alcohol 4 h,85% alcohol 2 h,90% alcohol 2 h,95% alcohol 1 h, anhydrous ethanol I 30 min, anhydrous ethanol II 30 min, alcohol benzene 5–10 min, xylene II 5–10 min,65°C melting paraffin I 1 h, 65°C melting paraffin II 1 h,65°C melting paraffin III 1 h.
3) The wax-soaked tissue is embedded in the embedding machine. After the wax is solidified, the wax block is removed from the embedded frame and repaired.
4) Slice the modified tissue wax block. The slice thickness is 4 μm. After the water-baked dried wax is melted, then taked out and stored at room temperature.

### 2.8 Immunofluorescence

After behavioral tests, mice were perfused. They were sequentially perfused with 40 ml of normal saline and 40 ml of a 4% paraformaldehyde solution (0.1 mol/L PBS, pH 7.4) through the ascending aorta after anesthesia with chloral hydrate. The mouse brain was fixed in 4% paraformaldehyde solution for more than 24 h, then dehydrated in gradient alcohol. Brain slices (thickness: 4 µm) were cut using a Frozen platform (JB-L5, Wuhan Junjie Electronics Co., Ltd., China) and collected in an EDTA antigen retrieval solution (pH 8.0). Next, the brain slices were rinsed with PBS (pH 7.4) three times for 5 min, then placed in 3% BSA and incubated at room temperature for 30 min. The brain slices were transferred into PBST with a primary antibody (Anti-P2X7R, 1:200, Bioss; Anti-Iba1, 1:500, Servicebio; Anti-Brdu, 1:200; Servicebio) and incubated at 4°C overnight. After rinsing with PBS (pH 7.4) for 5 min three times, the brain slices were transferred into PBS (pH 7.4) with a secondary antibody (FITC, 1:400, Servicebio; Cy3, 1:400, Servicebio) and incubated in the dark at room temperature for 50 min. For immunofluorescence staining, the patches were rinsed three times with PBS for 5 min three times, then dried with a 4’, 6-diamidino-2-phenylindole staining solution. Images were obtained using an Ortho-Fluorescent Microscopy (Nikon Eclipse C1, Nikon, Japan).

### 2.9 Western blot analyses

First, the hippocampus was lysed using a lysis buffer (RIPA buffer with proteinase inhibitors). Then, samples were quantified by the BCA protein quantitative detection kit before Western blotting. Equal amounts of total protein extracts were separated by SDS-PAGE and electro-transferred to PVDF membranes (0.45 μm). Then, the anti-NLRP3 and anti-P2X7 was used as the primary antibody, followed by incubation with appropriate HRP-conjugated secondary antibodies (GB23303, Servicebio). Proteins were detected with ECL (G2014, Servicebio) and quantified by densitometry using analytic software (alphaEaseFC, Alpha Innotech). Results were normalized using anti-β-actin antibody densitometric values.

### 2.10 Enzyme-linked immunosorbent assay

The concentration of serum and hippocampus NLRP3 and inflammatory cytokines IL-1β was measured using ELISA. First, the ELISA kits (Wuhan Genemei Biotechnology Co., Ltd., Wuhan, China) were brought to room temperature for 10–20 min. Standard and blank solutions were added into appropriate microwells before the test samples. After covering with plate membranes, the enzyme marker plates were incubated at 37°C for 30 min, then quickly washed five times. Next, the detection reagent and substrate solution were added into microwells according to the manufacturer’s instructions. Finally, the absorbance was detected at 450 nm immediately after the reaction was stopped with 50 µl of stop solution.

### 2.11 Immunohistochemistry analyses

Immunosuppression (IHC) analyses were performed on tissue sections (4 μm) obtained from mice groups. Briefly, slices were deparaffinized, and antigen retrieval was performed in a Microwave oven containing EDTA (pH 6.0) for 23 min. Rabbit anti-Iba1 (GB13105, Servicebio) antibodies (1:2500) were added and incubated with the tissues overnight at 4°C. Further, the universal secondary antibody (Servicebio) was incubated with the tissues for 50 min at room temperature. Diaminobenzidine was used as the chromogen, and, before mounting, slices were counterstained with hematoxylin. The study was approved by the Ethics Committee of the Shandong University of Traditional Chinese Medicine.

### 2.12 BrdU staining

Three days before sampling, each mice group was intraperitoneally injected with BrdU solution. The BrdU powder was dissolved in physiological saline, and the injection dose was 100 mg/kg for three consecutive days, twice a day, with 2-h intervals. After behavioral experiments, the brains of each mice group were perfused and placed in EP tubes with 4% paraformaldehyde for fixation. Fixed tissue samples were sectionized with paraffin, and immunofluorescence was finally detected.

### 2.13 HE staining

First, samples were dewaxed as follows: Xylene I for 20 min; Xylene II for 20 min; 100% ethanol I for 5 min; 100% ethanol II for 5 min; 75% ethanol for 5 min. Then, they were rinsed with tap water and the sections were stained with Hematoxylin solution for 3–5 min and rinsed again with tap water. Next, sections were treated with the Hematoxylin Differentiation solution and rinsed with tap water. The section was treated with Hematoxylin Scott Tap Bluing, rinsed with tap water, 85% ethanol for 5 min, and 95% ethanol for 5 min. Finally, sections were stained with Eosin for 5 min and dehydrated as follows: 100% ethanol I for 5 min; 100% ethanol II for 5 min; 100% ethanol III for 5 min; Xylene I for 5 min; Xylene II for 5 min. Finally, samples were sealed with neutral gum and observed under the microscope. Images were acquired and further analyzed.

### 2.14 Transmission electron microscope

Transmission electron microscopy was performed on hippocampus isolated and fixed with TEM Fixative. Samples were placed on 1% OsO_4_ (Ted Pella Inc., California, United States) in 0.1 M PBS (pH 7.4) for 2 h at room temperature. Then, tissues were dehydrated at room temperature, and, after resin penetration and embedding, tissues were stained with 2% uranium acetate saturated alcohol solution and 2.6% lead citrate. Finally, they were observed using a Transmission Electron Microscope (HT7800, Hitachi, Japan).

### 2.15 Data analyses

Statistical analyses were performed using GraphPad Prism 8 (GraphPad Software Inc., La Jolla, CA). Data are presented as means ± SEMs. One-way ANOVA was used to compare differences among groups, and a *p* < 0.05 was considered statistically significant.

## 3 Results

### 3.1 Intervention of the P2X7R-NLRP3-IL-1β pathway can ameliorates the resident intruder paradigm-induced aggressive behavior of mice

**FIGURE 1:**
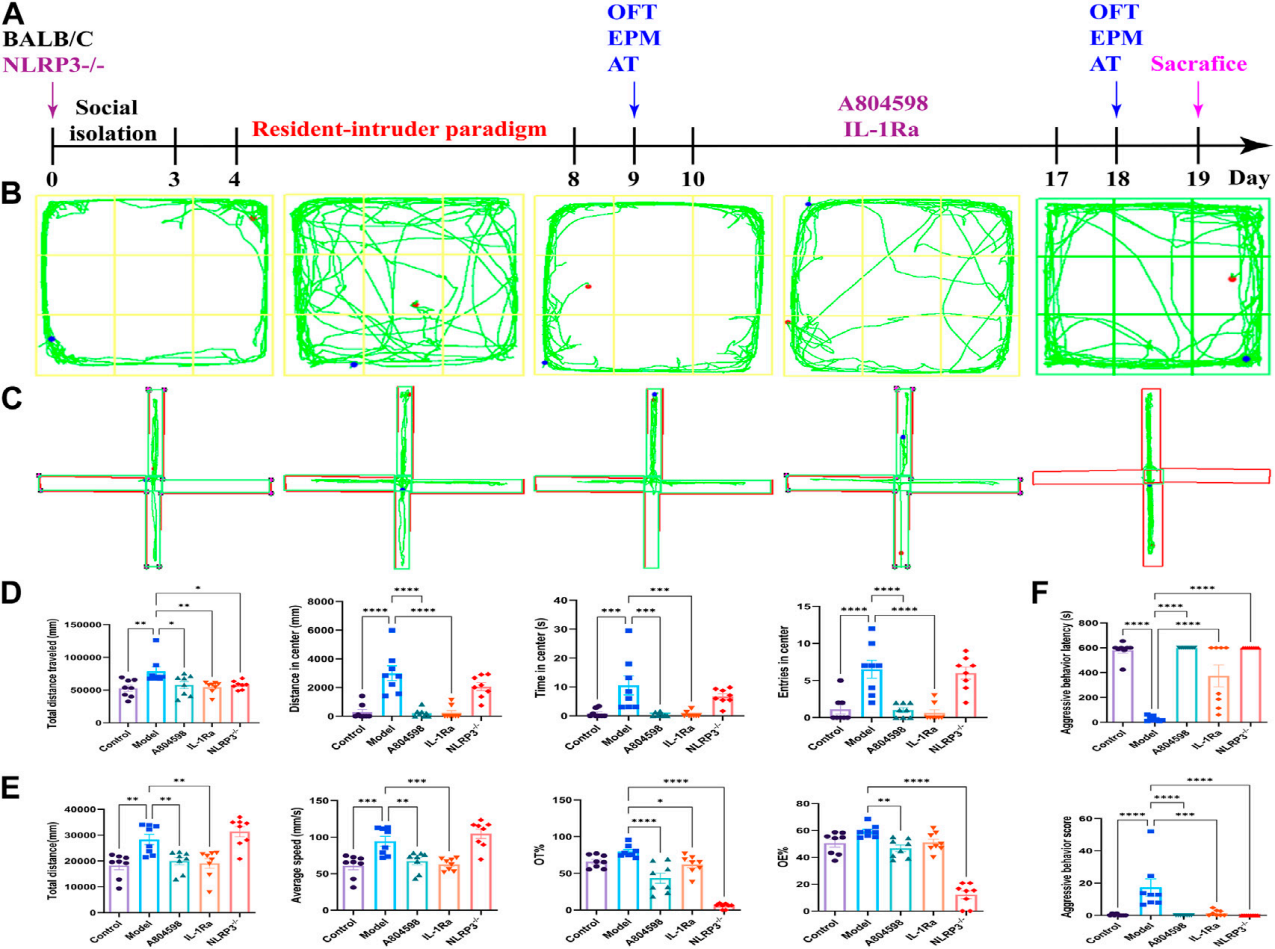
Behavioral phenotype of mice in each group. **(A)** Schematic representation of the experimental design. **(B)** Open field test representative trajectory graph. **(C)** Elevated plus-maze test representative trajectory graph. **(D)** Open field test results. **(E)** Elevated plus-maze test results. **(F)** Attack behavior test results. Data are expressed as means ± SEMs. *p < 0.05, **p < 0.01, ***p < 0.001, ****p < 0.0001. Control, producers of non-aggressive behavior after stress; Model, aggressive behavior after stress; A804598, P2X7R antagonist; IL-1Ra, IL-1β blocker; NLRP3^−/−^, NLRP3 knockout mice. *n* = 8/group. (TIFF format, 1200 dpi, 2-column fitting image).

To investigate aggressive behaviors, OFT, EPM, and AT were performed on the day after the last dose of treatments (Figure 1A). In OFT, model mice showed signs of increased excitability with a significantly increased distance traveled and time spent in the center area, as well as an increased number of entries into the central squares. Model mice treated with A804598 or IL-1Ra and knock out NLRP3 showed decreased excitability-like behaviors and decreased travel distance, residence time, and entries into the center of the arena (Figures 1B, D). In the EPM, the percentage of time and entries in the open arms was significantly increased in model mice compared to controls. Moreover, A804598 or IL-1Ra-treated and NLRP3^−/−^ mice exhibited decreased open arm exploration (Figures 1C, E). Then, the aggressive behavior level of mice in each group was measured using AT. Compared to Control mice, the resident-intruer paradigm significantly decreased aggressive behavior latency and increased score levels. Meanwhile, NLRP3^−/−^, A804598, or IL-1Ra treatments altered aggressive behavior latency and score levels compared to Model mice (Figure 1F).

### 3.2 The P2X7R-NLRP3-IL-1β pathway was activated in resident-intruder paradigm

**FIGURE 2:**
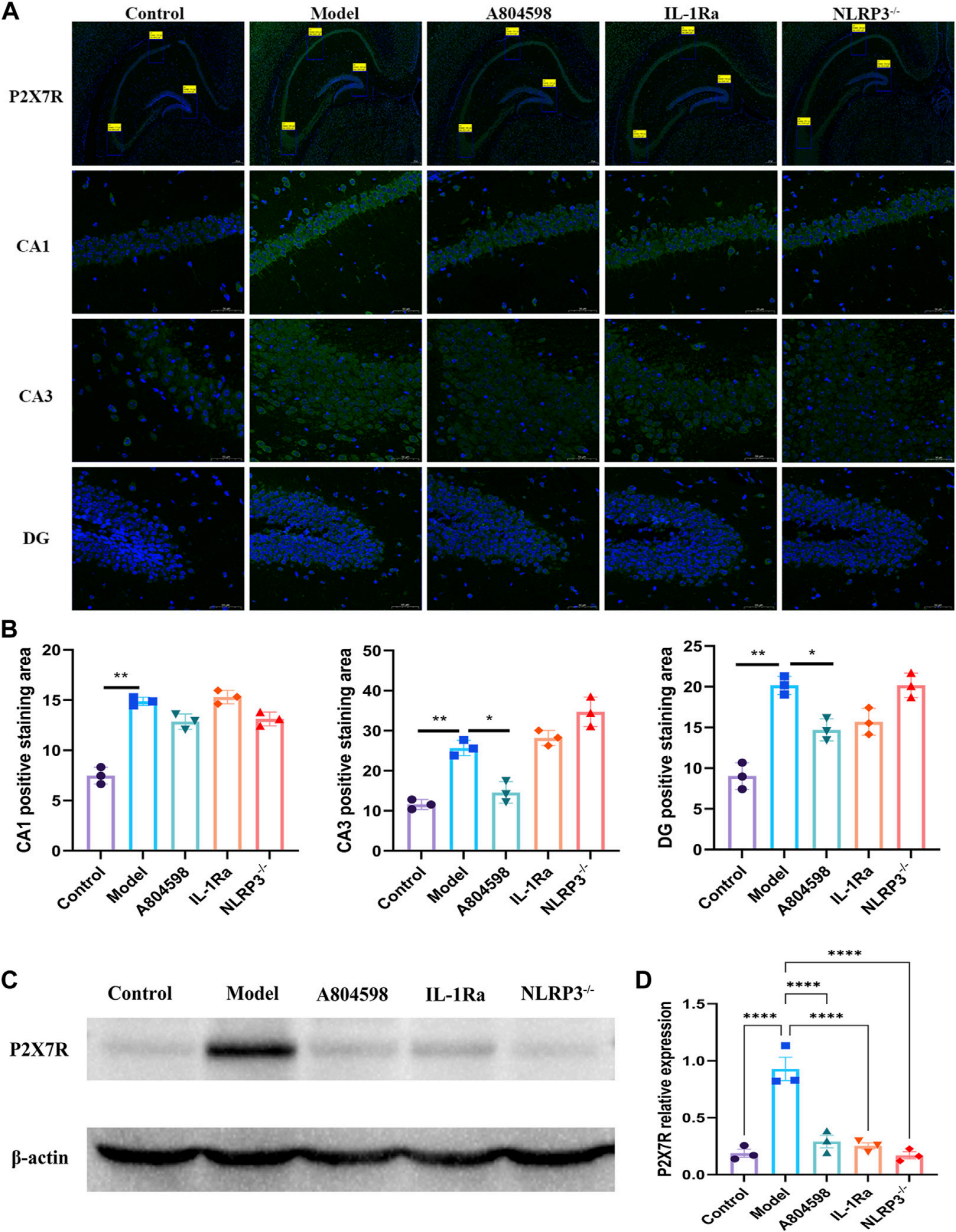
The P2X7R was activated in the resident intruder paradigm. **(A)** Representative images of P2X7R (green) and DAPI (blue) labeling in the hippocampus CA1, CA3, and DG after drug interventions; scale bar = 200 and 50 µm. **(B)** Positive staining area of P2X7R and DAPI double-labeled (P2X7R + DAPI) proteins in the hippocampus CA1 (**p < 0.01 _Control *vs.* Model_; *n* = 3/group), CA3 (**p < 0.01 _Control *vs.* Model_; *p < 0.05 _A804598 *vs.* Model_; *n* = 3/group), and DG (**p < 0.01 _Control *vs.* Model_; *p < 0.05 _A804598 *vs.* Model_; *n* = 3/group). **(C)** Representative immunoreactive bands showing the protein levels of hippocampal P2X7R in the Control, Model, A804598, IL-1Ra, and NLRP3^−/−^ mice. **(D)** Statistical results show that A804598, IL-1Ra, and NLRP3^−/−^ decreased the protein expression of P2X7R (*n* = 3, ****p < 0.0001 *vs.* Model).

**FIGURE 3:**
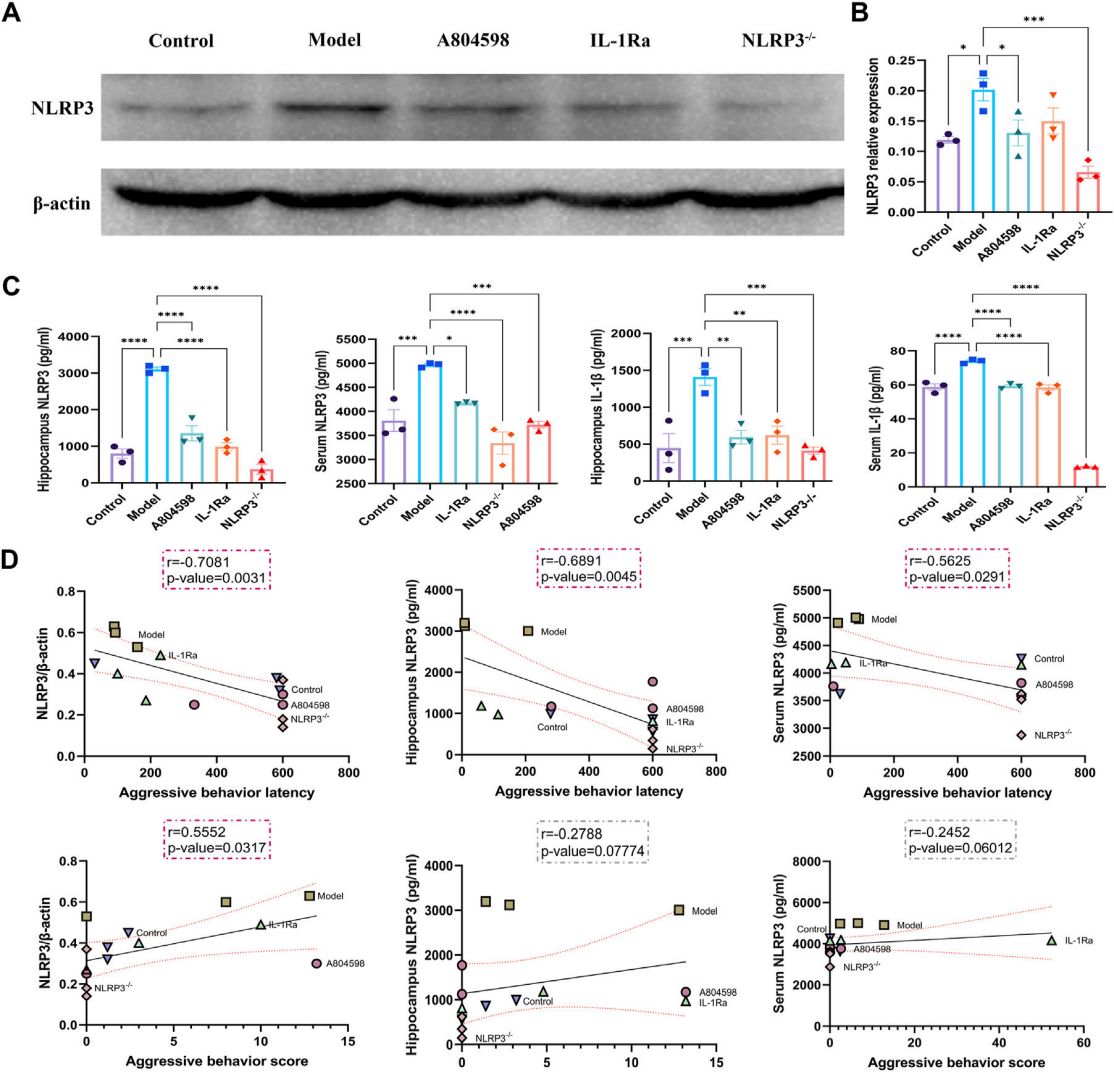
Correlations between the expression levels of NLRP3 in the hippocampus or serum and aggressive behaviors. **(A)** Representative immunoreactive bands showing the protein levels of hippocampal NLRP3 in the Control, Model, A804598, IL-1Ra, and NLRP3^−/−^ mice. **(B)** Statistical results show that A804598, IL-1Ra, and NLRP3^−/−^ decreased the protein expression of NLRP3 (*n* = 3, ****p < 0.0001 vs. Model). **(C)** NLRP3 and IL-1βhippocampus and serum levels. **(D)** Pearson correlation analyses were performed by comparing the expression levels of NLRP3 in the hippocampus or serum and aggressive behavior latency or aggressive behavior score. Correlations were performed considering some animals (*N* = 3). The coefficient *r* and *p*-value for each correlation are presented in a box. Statistically significant correlations are highlighted in red (*p* < 0.05). Data are expressed as means ± SEMs. Control, producers of non-aggressive behavior after stress; Model, aggressive behavior after stress; A804598, P2X7R antagonist; IL-1Ra, IL-1β blocker; NLRP3^−/−^, NLRP3 knockout mice. (TIFF format, 1200 dpi, 2-column fitting image).

After behavioral experiments, the hippocampus of mice was collected to detect the activation of the P2X7R-NLRP3-IL-1β pathway. Immunofluorescence results showed that P2X7R protein levels in hippocampus CA3 and DG in the Model group increased compared to Control (*p* = 0.0050, *p* = 0.0020) and A804598 (*p* = 0.0138, *p* = 0.0184) groups. The P2X7R protein levels in hippocampus CA1 in the Model group also increased compared to the Control group (*p* = 0.0024, Figure 2A, B). Our data showed that stress stimuli significantly increased the protein expression of P2X7R (Figures 2C, D), indicating P2X7R was activated in stress induced aggressive model. To evaluate the mechanisms of attack associated with the NLRP3 inflammasome, the NLRP3 proteins, activated by the P2X7R pathways, were analyzed in each group. The NLRP3 inflammasome was detected in mice’s hippocampus and β-actin was used as an NLRP3 negative marker (Figures 3A, B). These results demonstrated that the resident intruder paradigm can induce a significant increase in NLRP3 inflammasome (*p* = 0.0393 Control vs*.* Model). Significant differences were also found between A804598 (*p* = 0.0054), NLRP3^−/−^ (*p* = 0.0046), and Model mice. Then, the serum and hippocampus levels of NLRP3 and IL-1β were measured by ELISA. Compared to the Control group, the resident intruder paradigm significantly increased NLRP3 and IL-1β levels, while A804598, IL-1Ra, and NLRP3^−/−^ treatments altered NLRP3 and IL-1β levels compared to Model mice (Figure 3C). At the same time, in the hippocampus and serum, NLRP3 was negatively correlated with the aggressive behavior latency (aggressive behavior latency *vs.* NLRP3/β-actin: *r* = 0.7081, *p* = 0.0031; aggressive behavior latency *vs.* hippocampus NLRP3: *r* = 0.6891, *p* = 0.0045; aggressive behavior latency *vs.* serum NLRP3: *r* = 0.5625, *p* = 0.0291; Figure 3D), in the hippocampus, NLRP3 was positively correlated with the aggressive behavior score (aggressive behavior score *vs.* NLRP3/β-actin: *r* = 0.5552, *p* = 0.0317; Figure 3A), suggesting that increased NLRP3 expression levels in the hippocampus and serum might contribute to or be a consequence of aggressive behaviors.

### 3.3 Microglia were markedly activated and nerve regeneration was markedly reduced in aggressive behavior mice

**FIGURE 4:**
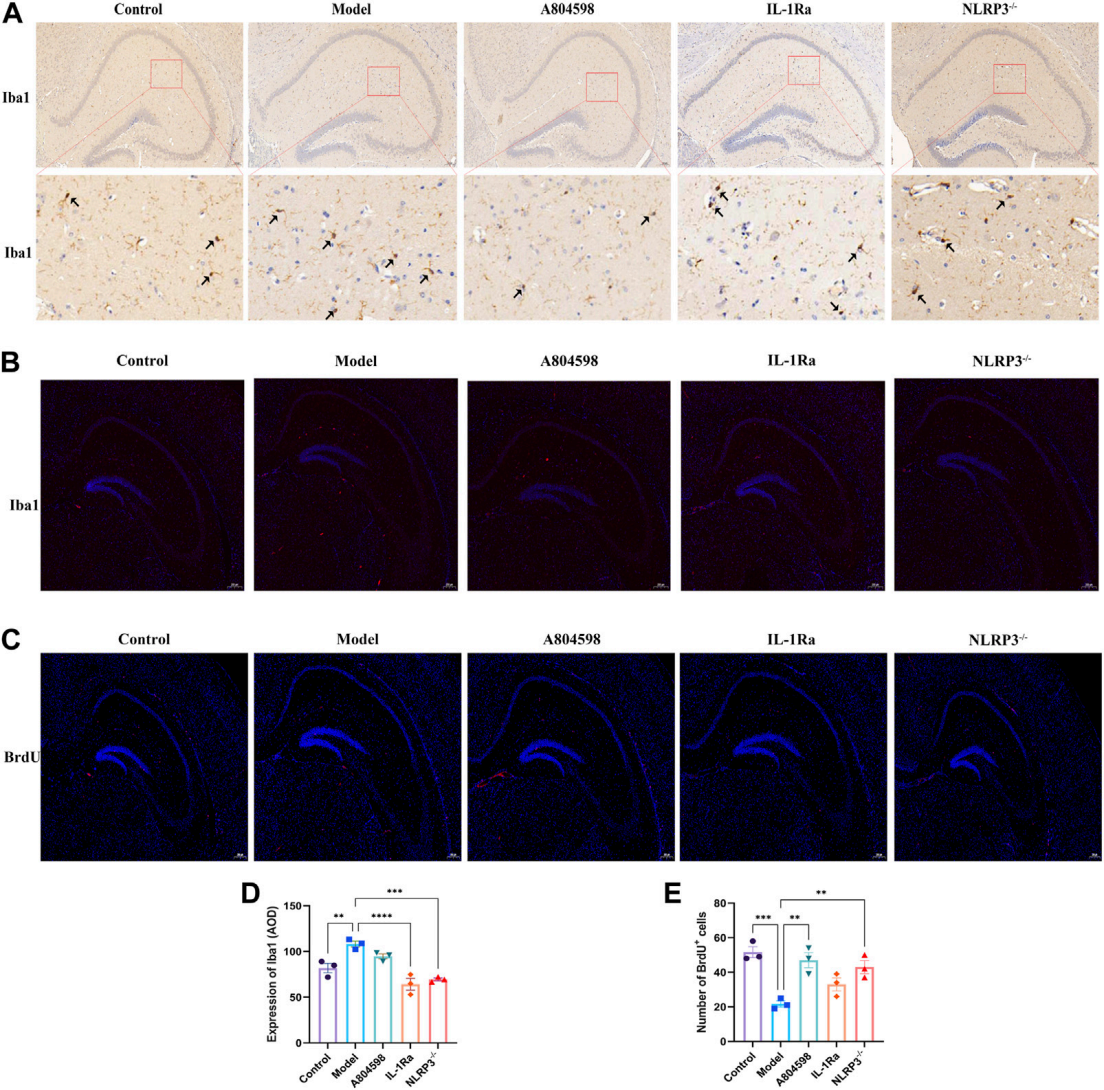
Microglia activation (Iba1) and Nerve regeneration (BrdU) in the hippocampus tissue of each mice group. **(A)** Immunohistochemistry (IHC) images for Iba1. **(B)** Representative images of Iba1 (red) and DAPI (blue) labeling in the hippocampus after drug interventions; scale bar = 200 µm. **(C)** Representative images of BrdU (red) and DAPI (blue) labeling in the hippocampus after drug interventions; scale bar = 200 µm. **(D)** Quantification of Iba1 IHC results in the hippocampus tissue of each mice group. The Iba1 protein levels were quantified according to the Average Optical Density (AOD). Iba1 protein levels were markedly increased in Model *versus* Control hippocampus tissues. **p < 0.01, ***p < 0.001 and ****p < 0.0001, compared with normal tissues. **(E)** Number of BrdU and DAPI double-labeled (BrdU + DAPI) neurons in the hippocampus (***p < 0.001 _Control vs. Model_; **p < 0.01 _A804598 vs. Model_; **p < 0.01 _NLRP3_^−/−^_vs. Model_; *n* = 3/group).

The IHC results revealed that microglia were markedly activated in Model hippocampus specimens compared to Control tissues (Figures 4A, D). After behavioral experiments, mice’s hippocampus were retrieved to detect microglia activation and nerve regeneration. The immunofluorescence results showed that Iba1 protein levels in the Model group hippocampus increased compared to Control, IL-1Ra, and NLRP3^−/−^ groups (Figure 4B). The number of BrdU proteins in the Model group hippocampus also reduced compared to Control (*p* = 0.0004), A804598 (*p* = 0.0015) and NLRP3^−/−^ (*p* < 0.0050) groups (Figures 4C, E).

### 3.4 Pathological and ultrastructural characterization of hippocampus

**FIGURE 5:**
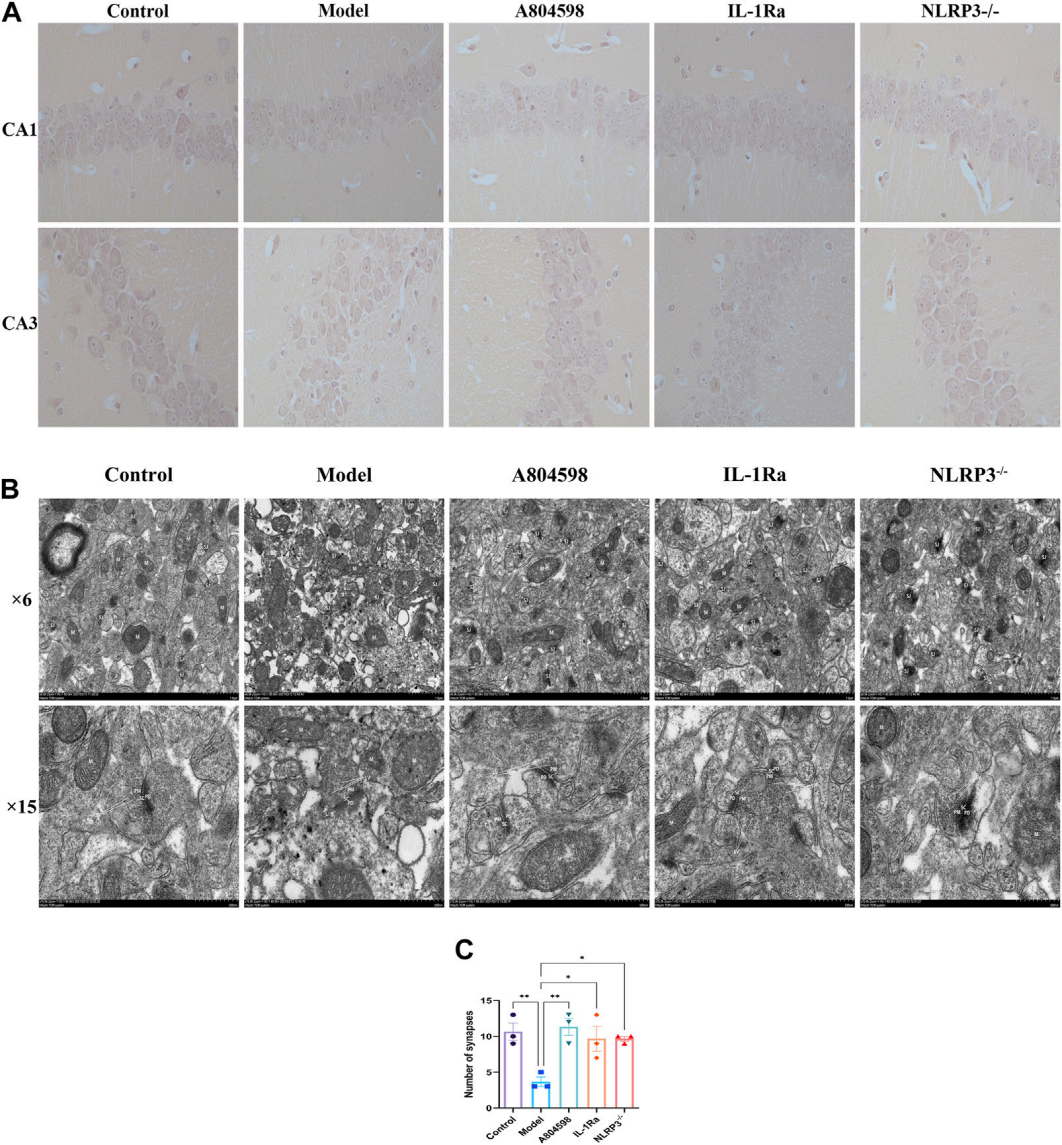
Pathological and ultrastructural characterization of the hippocampus of each mice group. **(A)** Drugs effects on the morphological structure of hippocampal CA1 and CA3 areas in aggressive behavior mice. **(B)** TEM representative images of the hippocampus of each mice group. Original magnification 6 K and 15 K, scale bar: 1 μm and 500 nm. Synapse (SJ), mitochondria (M), synaptic space (SC), presynaptic membrane (PM), postsynaptic membrane (PD), synaptic vesicles (SV), axonal terminals (T), dendritic spines (S). **(C)** Number of synapses in hippocampus of mice in each group (*n* = 3/group).

The nerve cells in the hippocampus CA1 and CA3 regions of the Control mice were arranged tightly, neatly, and presented with clear nuclei (Figure 5A). The nerve cells of the Model group were scattered, the cell volume was smaller and with a lot of degeneration. Degenerated nerve cells were darker, the nucleus pyknotic, and the cell body irregular. Moreover, each administration group significantly improved the above-mentioned pathological damages. The improvement effect of the A804598 group was equivalent to the Control group. Additionally, the hippocampus from all mice groups was analyzed using TEM (Figure 5B). In Control group, mitochondria (M) are mostly normal structurally, with intact membranes and cristae in the low power lens. The width of high power synaptic space (SC) is moderate, the gap boundary is obvious, the presynaptic membrane (PM) and postsynaptic membrane (PD) are complete and continuous, and the thickness of postsynaptic dense area is uniform. Synaptic vesicles (SV) are abundant. In model group, synaptic bodies edema and membrane damage, synaptic structure is seriously damaged with many vacuoles, mitochondria (M) membrane is blurred and crista is widened. High power synaptic space (SC) narrows, some visual field gaps are blurred, anterior and posterior membranes are coupled, gaps disappear, presynaptic membrane (PM) and posterior membranes (PD) exist, the structure is blurred, and the electron density of the postsynaptic dense area is high, complete and thick. Synaptic vesicles (SV) are abundant, mostly damaged, and their structures are incomplete; The axon terminal (T) has edema, accompanied by membrane damage and dissolution. Dendritic spines (S) also had edema, decreased electron density, and microfilament microtubules in both decreased and disappeared. After the intervention of A804598, IL-1Ra and NLRP3^−/−^, some synaptic bodies in the low power field of the neuropil became edema and the synapse was slightly damaged. Mitochondria (M) slightly swollen. The width of high power synaptic space (SC) is moderate. A small amount of visual field space is blurry. The presynaptic membrane (PM) and postsynaptic membrane (PD) are clear and partially damaged. The electron density of the postsynaptic dense area is high, clear and uniform. Synaptic vesicles (SV) were abundant and partially damaged. Compared to the Control group, the number of synapses in the hippocampus significantly reduced in the Model group (*p* = 0.0050). Compared to the Model group, the synapses significantly increased in the hippocampus of the A804598, IL-1Ra and NLRP3^−/−^ group (*p* = 0.0027, *p* = 0.0133, *p* = 0.0133) (Figure 5C).

## 4 Discussion

In the present study, we demonstrated that the NLRP3 inflammasome and its related inflammatory signaling pathways were activated in the resident intruder paradigm-induced mice model of aggressive behavior. Overall, blockage of NLRP3 inflammasome-driven inflammatory signaling ameliorated the aggressive behavior of mice, inhibited microglial activation and hippocampal ultrastructure damage, and promoted nerve regeneration. These results suggested that the NLRP3 inflammasome is involved in aggressive behaviors through P2X7R-NLRP3-IL-1β pathway regulation.

Para-inflammation is an immune state caused by stress or dysfunction and is considered an intermediate state between body homeostasis and classic inflammation (Medzhitov, 2008). Generally, inhibiting neuroinflammation can have therapeutic effects on neurodegenerative diseases. However, neuroinflammation can delay or aggravate the pathological changes of neurodegenerative diseases. The mechanisms of action are complex and many problems remain unsolved. The latest credible evidence showed that emotional stress can increase the level of peripheral inflammation (Shinobu et al., 2015), but the related inducing mechanism remained unclear. For example, in animal studies, it has been observed that the loss of the IL-18 gene can lead to aggressive behaviors in mice, providing clues about the etiological mechanisms of aggression (Lisboa et al., 2018). The relationship between C-reactive protein (CRP) and aggressive behaviors has been consistently confirmed in several studies and presented a positive correlation (Barzilay et al., 2016). For example, Orsolini et al. (2018) systematically assessed the association between CRP levels and schizophrenia severity (including effects on aggressiveness and impulsivity). Moreover, CRP levels were higher when tension characteristics, negative symptoms, and aggression, but did not seem to be related to suicidal behaviors. CoccaroRoyce and Mary (2014) also found that plasma CRP and IL-6 levels are directly related to aggressive behaviors. Previous autopsies found that the morphology and density of microglia neuroinflammation in the forehead and hippocampus of suicidal depression patients changes the distribution and function of stress-related immune cells, comprehending an important impact on the central inflammatory state (Torres-Platas et al., 2014). Also, repeated exposure to social frustration increases the accumulation of peripheral monocytes in the brain, leading to increased neuroinflammation and increased anxiety-like behaviors (Wohleb et al., 2014). During the resistance to the invasion of foreign substances, the NLRP3 inflammasome can recognize exogenous pathogen-associated molecular patterns (PAMPs) or endogenous damage-associated molecular patterns (DAMPs), and mediate the expression of inflammatory factors in downstream pathways through the activation of pattern recognition receptors (PRR), finally regulating inflammatory responses (Guo et al., 2015), and representing an important and inherent part of the body’s immune response. Recent studies Segovia et al. (2017) have shown that the abnormal activation of inflammasomes is related to the pathological response caused by innate immune disorders, playing an important role in many human immune diseases. The activated inflammasome complex processes procaspase-1 into active caspase-1 by catalytic cleavage, and catalyzes proIL-1β and pro-IL-18 into interleukin IL-1β with pro-inflammatory activity, IL-18 (Liu et al., 2014). Besides promoting the proliferation of macrophages, IL-1β can lead to the proliferation of neuroinflammation-related cells such as microglia and astrocytes (Feder and Laskin, 1994). When the central nervous system is damaged or inflammation occurs, these cells will be recruited to the corresponding parts to mediate the occurrence of neuroinflammation. At the same time, this mechanism is also related to different neurodegenerative diseases such as Alzheimer’s disease (AD) and Parkinson’s disease (PD) (Meyer-Luehmann et al., 2008). Moreover, the NLRP3 inflammasome plays an important role in these processes and can be a therapeutic target (Heneka et al., 2014). However, due to the incomplete study of its mechanisms, the simple application of NLRP3 inflammasome-related inhibitors might lead to systemic reactions and serious side effects (Gold and El Khoury, 2015). Therefore, future studies on new treatment methods need to focus on the specific pathways activated by NLRP3 in the central nervous system and explore possible related receptors or inhibitors. For example, in addition to lymphocyte proliferation and apoptosis, the purinoceptor P2X ligand-gated ion channel 7 (P2X7R) is an adenosine triphosphate (ATP)-gated receptor widely expressed in various immune cells, mainly through the activation of the NLRP3 inflammasome and the release of inflammatory cytokines involved in inflammation and autoimmune response (Federica et al., 2019). Clinical studies have found that the expression of the NLRP3 gene in peripheral blood mononuclear immune cells in depression patients is increased, as the corresponding IL-1β (Alcocer-Gómez et al., 2014). Additionally, blocking NLRP3 signaling can reduce hippocampal IL-1β levels and alleviate depression-like behaviors (Alcocer-Gómez et al., 2016). P2X7 antagonists can also block anhedonia and anxiety-like behaviors induced by chronic stress (Iwata et al., 2016). Therefore, we hypothesized that the P2X7R-mediated NLRP3 inflammasome was involved in the biological processes of aggressive behaviors. To verify this hypothesis, we used the resident intruder paradigm, previously established and published by our research group, to induce the aggressive behavior model, and the OFT, EPM, and AT to evaluate the model. However, in the OFT and EPM experiments, there was significant difference between the NLRP3 KO group and the IL-1Ra group. We speculate that this is due to the fact that different species of animals were used for the experiments. The mice used in the NLRP3 KO group were C57 and other groups were Balb/C. Generally, C57BL/6J mice had higher ambulatory movements than Balb/C (Rogers et al., 1999; Depino and Gross, 2007). In addition to augmented ambulatory activity, close arm duration was higher in C57BL/6J mice than Balb/C in OFT. (Lalonde and Strazielle, 2008). In addition, We found that the resident intruder paradigm intensified spontaneous movements and successfully induced aggressive behaviors in mice. For further confirmation, IF and Western blotting were used to detect P2X7R and NLRP3 inflammasome protein levels in the hippocampus of each mice group. Moreover, ELISA kits were used to detect NLRP3 and IL-1β in peripheral blood and hippocampus. The resident intruder paradigm increased P2X7R and NLRP3 inflammasome protein levels and promoted the release of IL-1β, finally leading to an inflammatory response. Also, the intervention on the P2X7R-NLRP3-IL-1β pathway reversed the neuroinflammation induced by resident intruder paradigm. These results indicated that the inflammatory pathology in peripheral blood and hippocampus triggered by P2X7R-NLRP3-IL-1β was essential for aggressive behaviors in mice.

Mental disorders characterized by persistent cognitive and behavioral symptoms are also associated with impaired neuroplasticity in several key cortical limbic brain regions. Recently, a neuroimaging study showed that the size of the prefrontal cortex and hippocampus of depressed patients was reduced. Besides, studies with animal models induced by psychological and environmental stress patterns provided direct evidence of neuroatrophy (Gálvez et al., 2017). The article “Frontiers in cellular neuroscience” also pointed out that neuroplasticity is the next direction for the development of antipsychotic drugs (Gálvez et al., 2017). Our previous research also found that hippocampal synaptic connections were reduced and neuronal morphology and function changed in the aggressive behavior model induced by the resident intruder paradigm (Peng et al., 2016). To study the relationship between neural plasticity damage caused by this paradigm and NLRP3, we used microglial cell-specific protein antibody Iba1 to specifically label microglial cells for IHC tests, and the ImageJ visualization software to analyze the activation of microglial cells. Additionally, we used an electron microscope to observe changes in hippocampal synapses and stained hippocampal slices with BrdU to observe the impact of residential invasion on hippocampal regeneration. It must be pointed out that we don't have clarified the cell type of BrdU positive cells. However, BrdU-positive cells in the hippocampus decreased compared to the control group in chronically stressed mice, and BrdU positive cells co-expressed the mature neuron marker NSE (Zuo et al., 2018). Meanwhile, one study found that antagonist of P2X7R have notably increased DCX level and the number of BrdU-tagged cells (Yun-Feng et al., 2010). We, therefore, speculative mostly that BrdU approximately tagged in neurons in this study, and block P2X7R-NLRP3-IL-1β pathway increases neurogenesis in resident-intruder paradigm. Therefore, the resident intruder paradigm-induced inflammatory process was crucial in the development of aggressive behaviors in mice. Moreover, NLRP3 can lead to neuroplastic damage, comprehending the initiating factor of aggressive behaviors.

## 5 Conclusion

In the present study, we demonstrated that NLRP3 inflammasome-driven inflammatory process is crucial in resident intruder paradigm-induced aggressive behavior mice. Additionally, these effects were mediated by the P2X7R-NLRP3-IL-1β pathway. Therefore, the NLRP3 inflammasome can be a potential anti-aggressive target, and its inhibition can protect against stress-induced aggressive behaviors.

Nevertheless, it should also be noted whether the therapeutic target is applicable to human beings requires to be further verified. Additional studies have also suggested to compare the changes in the expression of NLRP3 inflammatory bodies in the same animal before and after drug intervention in order to further confirm that the residential invasion paradigm leads to aggressive behavior by changing the expression of NLRP3 inflammatory bodies. Moreover, thefindings provided in this study are preliminary, indicating more studies in this area are required in the future. In the future, microdialysis sampling can be carried out on the hippocampus of model mice to study the impact of residential invasion paradigm on the release of inflammatory factors in the hippocampus.

## Data Availability

The original contributions presented in the study are included in the article/Supplementary Material, further inquiries can be directed to the corresponding authors.

## Supplementary material

Supplementary Figure S1 The results of behavioral test of mice in each group before administration.

Supplementary Figure S2 P2X7R and DAPI fluorescence staining were performed in the hippocampus of mice in each group.

Supplementary Figure S3 Single channel immunofluorescence map of Iba1 and BrdU in hippocampus of mice in each group.

Supplementary Figure S4 NLRP3: WB full gel.

Supplementary Figure S5 P2X7R: WB full gel.

## Abbreviations

NLRP3, nucleotide-binding oligomerization domain-like receptor protein 3; OFT, open-field test; EPM, elevated plus maze experiment; AT, aggressive behavior test; P2X7R, Purinoceptor P2X ligand-gated ion channel 7; ELISA, enzyme-linked immunosorbent assay; BrdU, 5-Bromodeoxyuridinc; IL-1β, interleukin-1β; IL-1Ra, interleukin-1 receptor antagonist; CRP, C-reactive protein; IL-6, interleukin- 6; C57BL/6, C57 black 6; EDTA, Ethylene Diamine Tetraacetic Acid; BSA, Bovine albumin; HRP, Horseradish Peroxidase; BCA, bicinchoninic acid; PVDF, poly (1,1-difluoroethylene); TBST, TBS + Tween; PBS, phosphate-buffered saline; EP, Eppendorf; SEM, Standard Error of Mean; ANOVA, Analysis of Variance; PAMPs, pathogen-associated molecular patterns; DAMPs, damage-associated molecular patterns; PRR, pattern recognition receptors; AD, Alzheimer’s disease; PD, Parkinson’s disease; ATP, adenosine triphosphate.
