# Supplementary figures and images for: The NLRP3 inflammasome is involved in resident intruder paradigm-induced aggressive behaviors in mice
Source: Front Pharmacol. 2023 Jan 25;14:974905. doi: 10.3389/fphar.2023.974905 (PMC9912938; doi:10.3389/fphar.2023.974905)

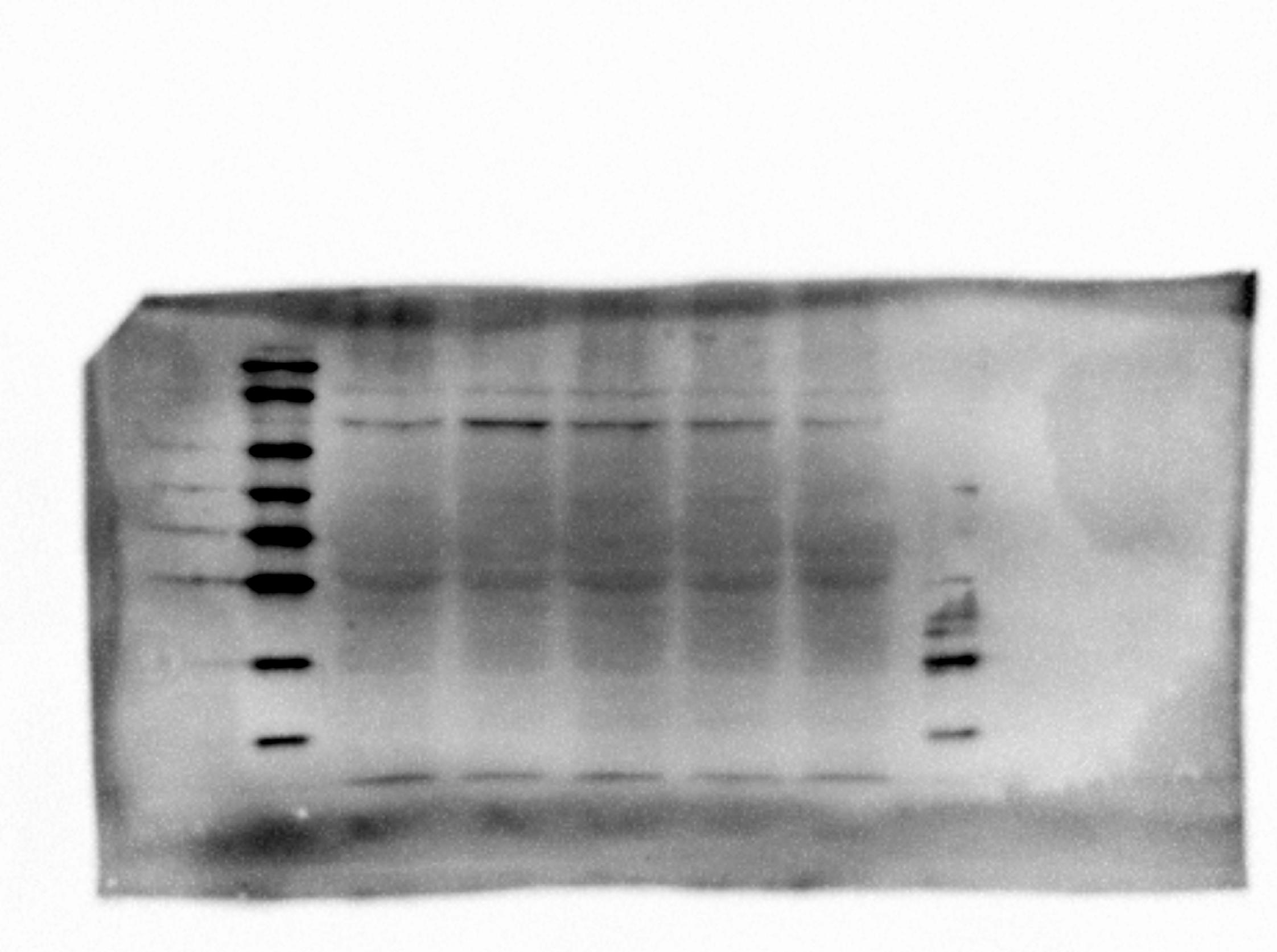

Supplement: Supplementary file 2 [file Image4.JPEG]

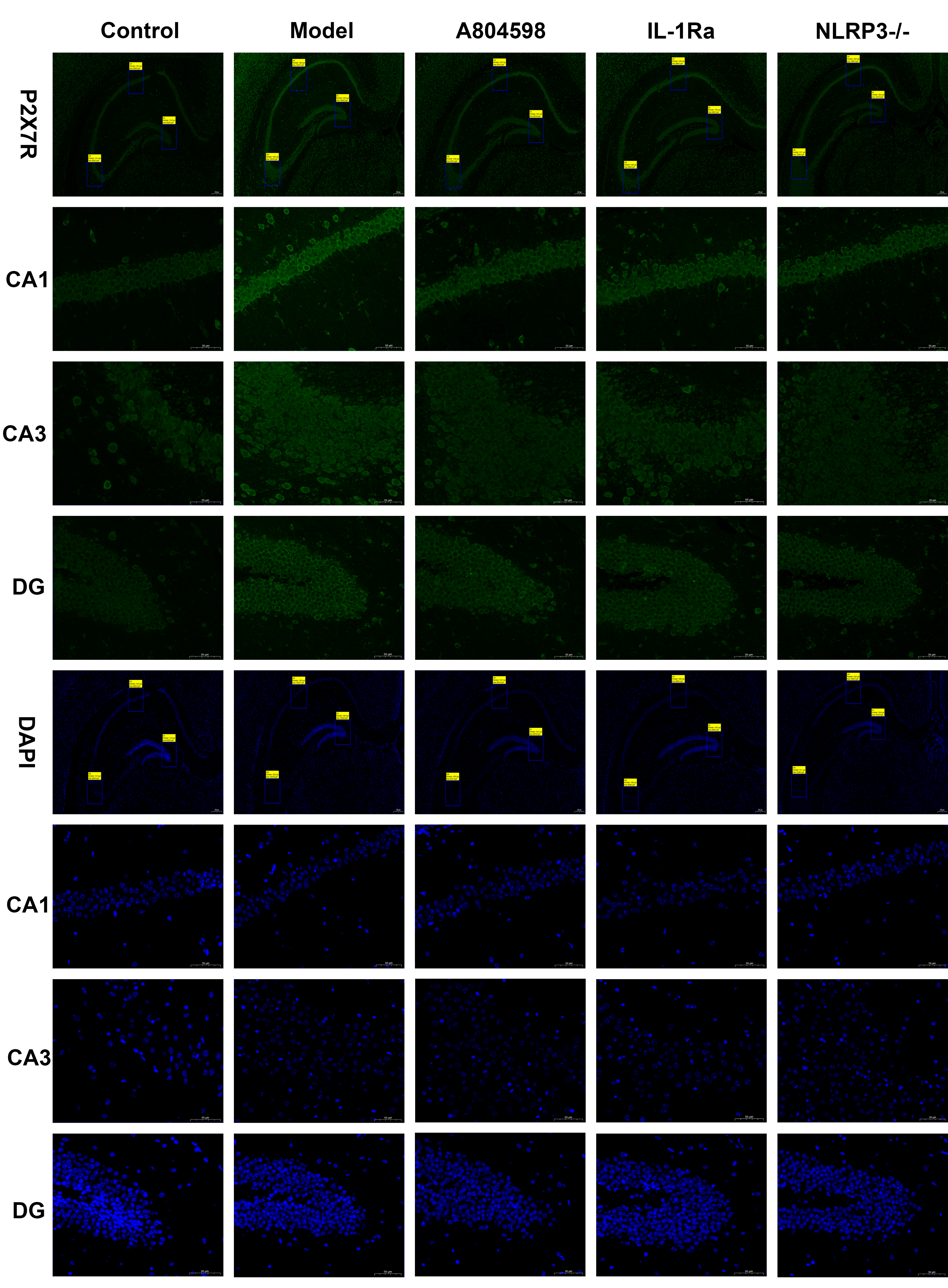

Supplement: Supplementary file 3 [file Image2.JPEG]

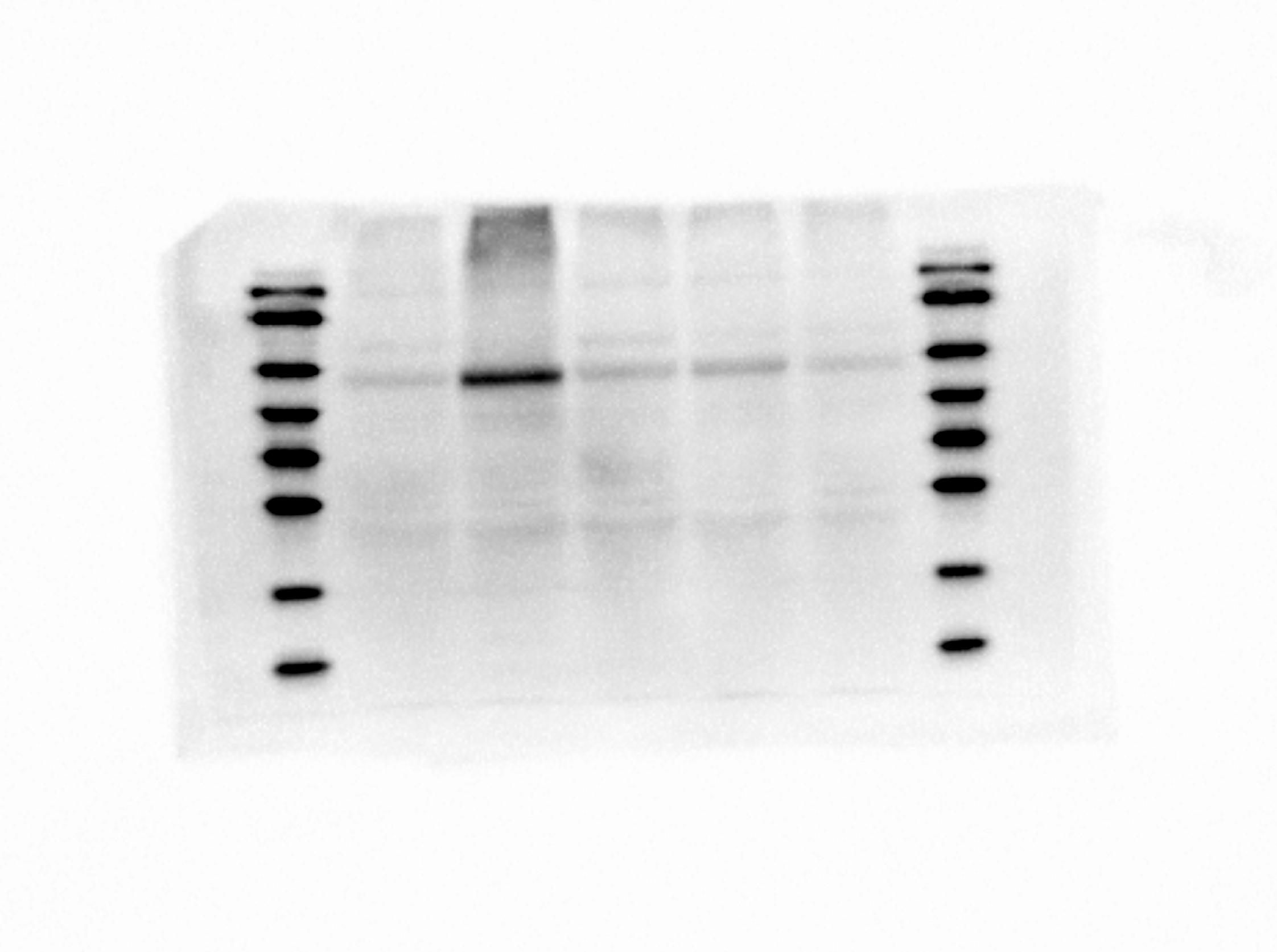

Supplement: Supplementary file 4 [file Image5.JPEG]

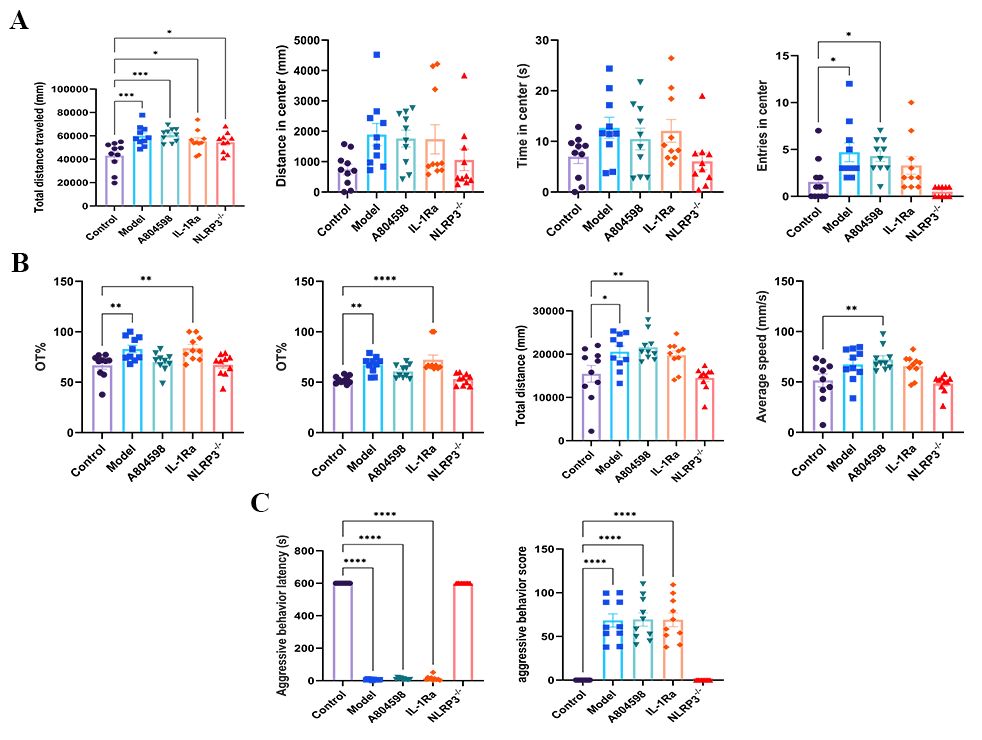

Supplement: Supplementary file 5 [file Image1.TIF]
